# 4-Amino­pyridinium 4-carb­oxy­butano­ate

**DOI:** 10.1107/S1600536810028734

**Published:** 2010-07-24

**Authors:** Hoong-Kun Fun, Madhukar Hemamalini, Venkatachalam Rajakannan

**Affiliations:** a X-ray Crystallography Unit, School of Physics, Universiti Sains Malaysia, 11800 USM, Penang, Malaysia; b Biomedical Structural Biology, School of Biological Sciences, Nanyang Technological University, Singapore 138673

## Abstract

The asymmetric unit of the title salt, C_5_H_7_N_2_^+^·C_5_H_7_O_4_^−^, contains two 4-amino­pyridinium cations and two 4-carb­oxy­butano­ate anions. Each 4-amino­pyridinium cation is planar, with a maximum deviation of 0.005 (2) Å. Both 4-carb­oxy­butano­ate anions adopt an extended conformation. In the crystal structure, the cations and anions are linked *via* N—H⋯O, O—H⋯O and C—H⋯O hydrogen bonds, forming a two-dimensional network parallel to the *bc* plane.

## Related literature

For the biological activity of 4-amino­pyridine, see: Schwid *et al.* (1997 ▶). For crystal structure determinations of 4-amino­pyridine, see: Chao & Schempp (1977 ▶); Anderson *et al.* (2005 ▶). For conformations of 4-carb­oxy­butano­ate (hydrogen glutarate) anions, see: Saraswathi *et al.* (2001 ▶).
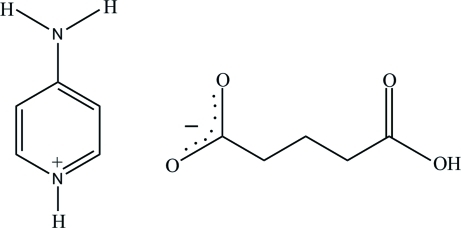


## Experimental

### 

#### Crystal data

C_5_H_7_N_2_^+^·C_5_H_7_O_4_^−^
*M* *_r_* = 226.23
Monoclinic, 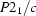

*a* = 9.6159 (2) Å
*b* = 10.3065 (2) Å
*c* = 22.6801 (5) Å
β = 102.143 (1)°
*V* = 2197.45 (8) Å^3^
*Z* = 8
Mo *K*α radiation
μ = 0.11 mm^−1^
*T* = 296 K
0.35 × 0.26 × 0.21 mm

#### Data collection

Bruker SMART APEXII CCD area-detector diffractometer
Absorption correction: multi-scan (*SADABS*; Bruker, 2009 ▶) *T*_min_ = 0.963, *T*_max_ = 0.978
7931 measured reflections
7931 independent reflections
5556 reflections with *I* > 2σ(*I*)

#### Refinement

*R*[*F* ^2^ > 2σ(*F* ^2^)] = 0.053
*wR*(*F* ^2^) = 0.154
*S* = 1.04
7931 reflections
290 parameters
H-atom parameters constrained
Δρ_max_ = 0.37 e Å^−3^
Δρ_min_ = −0.20 e Å^−3^

### 

Data collection: *APEX2* (Bruker, 2009 ▶); cell refinement: *SAINT* (Bruker, 2009 ▶); data reduction: *SAINT*; program(s) used to solve structure: *SHELXTL* (Sheldrick, 2008 ▶); program(s) used to refine structure: *SHELXTL*; molecular graphics: *SHELXTL*; software used to prepare material for publication: *SHELXTL* and *PLATON* (Spek, 2009 ▶).

## Supplementary Material

Crystal structure: contains datablocks global, I. DOI: 10.1107/S1600536810028734/wn2401sup1.cif

Structure factors: contains datablocks I. DOI: 10.1107/S1600536810028734/wn2401Isup2.hkl

Additional supplementary materials:  crystallographic information; 3D view; checkCIF report

## Figures and Tables

**Table 1:** Hydrogen-bond geometry (Å, °)

*D*—H⋯*A*	*D*—H	H⋯*A*	*D*⋯*A*	*D*—H⋯*A*
O1*A*—H1*O*1⋯O1*B*^i^	0.89	1.57	2.4591 (17)	172
N1*A*—H1*AA*⋯O1*B*^ii^	0.86	2.59	3.183 (2)	127
N1*A*—H1*AA*⋯O2*B*^ii^	0.86	1.87	2.729 (2)	177
N2*A*—H2*AC*⋯O2*A*^iii^	0.86	2.18	2.9627 (19)	151
N2*A*—H2*AD*⋯O4*B*	0.86	2.10	2.948 (2)	170
O3*B*—H1*O*3⋯O3*A*^iv^	0.92	1.56	2.4791 (17)	176
C10*A*—H10*A*⋯O4*B*^v^	0.93	2.55	3.161 (2)	124
C7*A*—H7*AA*⋯O3*B*	0.93	2.47	3.370 (2)	162

Symmetry codes: (i) 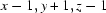; (ii) 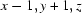; (iii) 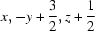; (iv) 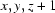; (v) 
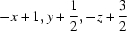
.

**Fig. 1.**
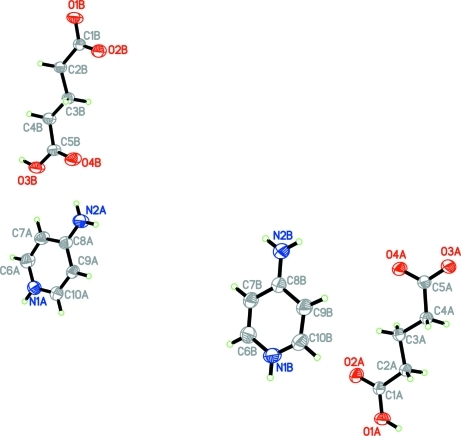
The asymmetric unit of the title compound. Displacement ellipsoids are drawn at the 50% probability level. Hydrogen atoms are shown as spheres of arbitrary radius.

**Fig. 2.**
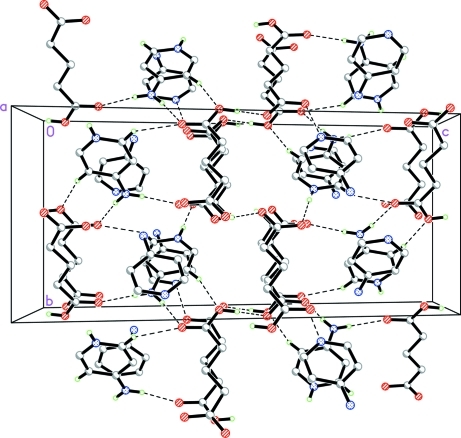
The crystal packing, showing hydrogen-bonded (dashed lines) 2D networks parallel to to the bc-plane. H atoms not involved in the intermolecular interactions have been omitted for clarity.

## supplementary crystallographic information

### Comment

4-Aminopyridine (fampridine) is used in the treatment of neurological ailments, such as multiple sclerosis (MS), with tests showing that fampridine improves motor function in MS patients (Schwid *et al.*, 1997). The room temperature crystal structure of 4-aminopyridine was reported many years ago (Chao & Schempp, 1977); a redetermination at low temperature has been reported more recently (Anderson *et al.,* 2005). Aliphatic dicarboxylic acids, HOOC(CH_2_)_n_COOH, exhibit interesting complexing properties. This is due to the flexibility of the ligand which may be completely or only partially deprotonated. Glutaric acid (1,3-propanedicarboxylic acid) is a white  crystalline solid which is very soluble in water. The compound is a useful building block for polymers, an intermediate in chemical synthesis and it is used in the manufacture of an antiretroviral drug. The compound is also used in solder flux. The present study has been carried out to study the hydrogen bonding patterns present in the crystal structure of 4-aminopyridinium 4-carboxybutanoate.

The asymmetric unit of the title compound consists of two crystallographically independent 4-aminopyridinium cations (A and B) and 4-carboxybutanoate (hydrogen glutarate) anions (A and B) (Fig. 1). Each 4-aminopyridinium cation is planar, with a maximum deviation of 0.005 (2) Å for atom C7A in cation A and 0.005 (2) Å for atom C9B in cation B. In the cations, protonation at atoms N1A and N1B lead to a slight increase in the C6A—N1A—C10A [120.06 (16)°] and C6B—N1B—C10B [120.21 (16)°] angles compared to those observed in the unprotonated structure of 4-aminopyridine (Anderson *et al.*, 2005). The conformations of the 4-carboxybutanoate anions can be described by the two torsion angles C1A—C2A—C3A—C4A of 175.44 (15)° and C2A—C3A—C4A—C5A of 174.42 (15)° in anion A; C1B—C2B—C3B—C4B of -175.99 (14)° and C2B—C3B—C4B—C5B of -176.94 (15)° in anion B. These torsion angles indicate that both anions adopt fully extended conformations (Saraswathi *et al.*, 2001).

In the crystal structure (Fig. 2), the cations and anions are linked via O1A—H1O1···O1B, N1A—H1AA···O1B, N1A—H1AA···O2B, N2A—H2AC···O2A, N2A—H2AD···O4B, O3B—H1O3···O3A, C10A—H10A···O4B and C7A—H7AA···03B intermolecular hydrogen bonds (Table 1), forming a two-dimensional network parallel to the *bc*-plane.

The crystal structure is a merohedral twin, with BASF = 0.3214 (15).

### Experimental

A hot methanol solution (20 ml) of 4-aminopyridine (0.0471 g, Aldrich) and glutaric acid (0.0661 g, Merck) was warmed for half an hour over a water bath. The mixture was cooled slowly and kept at room temperature. Colourless crystals were obtained after a few days.

### Refinement

Oxygen-bound H atoms were located in a difference map [O—H = 0.8903 and 0.9176 Å]. Nitrogen- and carbon-bound H atoms were positioned geometrically [N—H = 0.86 Å and C—H = 0.93–0.97 Å]. All hydrogen atoms were refined using a riding model, with *U*_iso_(H) = 1.2 *U*_eq_(C).

### Crystal data

**Table:**

C_5_H_7_N_2_^+^·C_5_H_7_O_4_^−^	*F*(000) = 960
*M**_r_* = 226.23	*D*_x_ = 1.368 Mg m^−^^3^
Monoclinic, *P*2_1_/*c*	Mo *K*α radiation, λ = 0.71073 Å
Hall symbol: -P 2ybc	Cell parameters from 8906 reflections
*a* = 9.6159 (2) Å	θ = 2.5–30.1°
*b* = 10.3065 (2) Å	µ = 0.11 mm^−^^1^
*c* = 22.6801 (5) Å	*T* = 296 K
β = 102.143 (1)°	Block, colourless
*V* = 2197.45 (8) Å^3^	0.35 × 0.26 × 0.21 mm
*Z* = 8	

### Data collection

**Table:**

Bruker SMART APEXII CCD area-detector diffractometer	7931 independent reflections
Radiation source: fine-focus sealed tube	5556 reflections with *I* > 2σ(*I*)
graphite	*R*_int_ = 0.0000
φ and ω scans	θ_max_ = 32.6°, θ_min_ = 1.8°
Absorption correction: multi-scan (*SADABS*; Bruker, 2009)	*h* = −14→14
*T*_min_ = 0.963, *T*_max_ = 0.978	*k* = −15→15
7931 measured reflections	*l* = −11→34

### Refinement

**Table:**

Refinement on *F*^2^	Primary atom site location: structure-invariant direct methods
Least-squares matrix: full	Secondary atom site location: difference Fourier map
*R*[*F*^2^ > 2σ(*F*^2^)] = 0.053	Hydrogen site location: inferred from neighbouring sites
*wR*(*F*^2^) = 0.154	H-atom parameters constrained
*S* = 1.04	*w* = 1/[σ^2^(*F*_o_^2^) + (0.0758*P*)^2^ + 0.2205*P*] where *P* = (*F*_o_^2^ + 2*F*_c_^2^)/3
7931 reflections	(Δ/σ)_max_ < 0.001
290 parameters	Δρ_max_ = 0.37 e Å^−^^3^
0 restraints	Δρ_min_ = −0.20 e Å^−^^3^

### Special details

**Table:**

Geometry. All esds (except the esd in the dihedral angle between two l.s. planes) are estimated using the full covariance matrix. The cell esds are taken into account individually in the estimation of esds in distances, angles and torsion angles; correlations between esds in cell parameters are only used when they are defined by crystal symmetry. An approximate (isotropic) treatment of cell esds is used for estimating esds involving l.s. planes.
Refinement. Refinement of F^2^ against ALL reflections. The weighted R-factor wR and goodness of fit S are based on F^2^, conventional R-factors R are based on F, with F set to zero for negative F^2^. The threshold expression of F^2^ > 2sigma(F^2^) is used only for calculating R-factors(gt) etc. and is not relevant to the choice of reflections for refinement. R-factors based on F^2^ are statistically about twice as large as those based on F, and R- factors based on ALL data will be even larger.

### Fractional atomic coordinates and isotropic or equivalent isotropic displacement parameters (Å^2^)

**Table:**

	*x*	*y*	*z*	*U*_iso_*/*U*_eq_	
O1A	0.28193 (16)	1.02435 (13)	0.06872 (6)	0.0516 (3)	
H1O1	0.2530	1.0161	0.0289	0.077*	
O2A	0.41099 (16)	0.95000 (13)	0.15319 (5)	0.0478 (3)	
O3A	0.76925 (16)	0.49292 (13)	0.05256 (5)	0.0528 (4)	
O4A	0.74003 (16)	0.55719 (12)	0.14167 (5)	0.0454 (3)	
C1A	0.3787 (2)	0.94695 (15)	0.09826 (7)	0.0352 (3)	
C2A	0.4485 (2)	0.85513 (16)	0.06135 (7)	0.0370 (3)	
H2AA	0.5028	0.9059	0.0381	0.044*	
H2AB	0.3745	0.8103	0.0330	0.044*	
C3A	0.5465 (2)	0.75453 (16)	0.09724 (7)	0.0352 (3)	
H3AA	0.6264	0.7976	0.1230	0.042*	
H3AB	0.4955	0.7064	0.1227	0.042*	
C4A	0.60070 (19)	0.66165 (16)	0.05536 (7)	0.0366 (3)	
H4AA	0.5202	0.6144	0.0322	0.044*	
H4AB	0.6415	0.7121	0.0271	0.044*	
C5A	0.7105 (2)	0.56458 (15)	0.08576 (7)	0.0346 (3)	
N1A	0.28175 (17)	0.87243 (15)	0.83697 (7)	0.0449 (4)	
H1AA	0.2264	0.9279	0.8486	0.054*	
N2A	0.53978 (18)	0.60387 (16)	0.78136 (7)	0.0499 (4)	
H2AC	0.5341	0.5918	0.7434	0.060*	
H2AD	0.5987	0.5590	0.8072	0.060*	
C6A	0.3744 (2)	0.80430 (19)	0.87789 (8)	0.0488 (4)	
H6AA	0.3778	0.8191	0.9186	0.059*	
C7A	0.4634 (2)	0.71465 (18)	0.86214 (8)	0.0461 (4)	
H7AA	0.5268	0.6692	0.8915	0.055*	
C8A	0.45783 (19)	0.69135 (17)	0.79991 (8)	0.0379 (3)	
C9A	0.3609 (2)	0.76547 (17)	0.75810 (7)	0.0399 (4)	
H9AA	0.3548	0.7543	0.7169	0.048*	
C10A	0.2754 (2)	0.85416 (19)	0.77826 (8)	0.0434 (4)	
H10A	0.2115	0.9027	0.7504	0.052*	
O1B	1.22169 (16)	−0.01002 (13)	0.95900 (5)	0.0527 (4)	
O2B	1.10552 (17)	0.05354 (13)	0.86994 (5)	0.0494 (3)	
O3B	0.72084 (16)	0.52422 (13)	0.94174 (6)	0.0532 (4)	
H1O3	0.7347	0.5145	0.9828	0.080*	
O4B	0.76125 (17)	0.44368 (13)	0.85744 (5)	0.0524 (3)	
C1B	1.12988 (19)	0.06276 (15)	0.92524 (7)	0.0359 (3)	
C2B	1.05243 (19)	0.16092 (15)	0.95600 (7)	0.0357 (3)	
H2BA	1.1223	0.2127	0.9831	0.043*	
H2BB	0.9966	0.1148	0.9803	0.043*	
C3B	0.9547 (2)	0.25132 (15)	0.91350 (7)	0.0342 (3)	
H3BA	0.8792	0.2016	0.8884	0.041*	
H3BB	1.0079	0.2948	0.8874	0.041*	
C4B	0.89090 (19)	0.35155 (16)	0.94935 (7)	0.0361 (3)	
H4BA	0.8441	0.3063	0.9773	0.043*	
H4BB	0.9677	0.4023	0.9731	0.043*	
C5B	0.7856 (2)	0.44294 (16)	0.91214 (7)	0.0358 (3)	
N1B	−0.04752 (17)	0.37734 (15)	0.17394 (7)	0.0453 (4)	
H1BA	−0.1137	0.4333	0.1619	0.054*	
N2B	0.26087 (18)	0.10594 (16)	0.23042 (7)	0.0494 (4)	
H2BC	0.2914	0.0928	0.2684	0.059*	
H2BD	0.2946	0.0613	0.2046	0.059*	
C6B	0.0019 (2)	0.35827 (19)	0.23332 (8)	0.0440 (4)	
H6BA	−0.0348	0.4067	0.2611	0.053*	
C7B	0.1058 (2)	0.26829 (17)	0.25317 (7)	0.0402 (4)	
H7BA	0.1392	0.2560	0.2943	0.048*	
C8B	0.16224 (19)	0.19448 (17)	0.21178 (7)	0.0377 (3)	
C9B	0.1092 (2)	0.21875 (18)	0.14972 (8)	0.0463 (4)	
H9BA	0.1444	0.1734	0.1206	0.056*	
C10B	0.0059 (2)	0.30944 (19)	0.13355 (8)	0.0494 (5)	
H10B	−0.0291	0.3250	0.0927	0.059*	

### Atomic displacement parameters (Å^2^)

**Table:**

	*U*^11^	*U*^22^	*U*^33^	*U*^12^	*U*^13^	*U*^23^
O1A	0.0584 (8)	0.0587 (8)	0.0349 (6)	0.0303 (7)	0.0034 (6)	−0.0017 (6)
O2A	0.0626 (9)	0.0535 (7)	0.0270 (5)	0.0165 (7)	0.0085 (6)	−0.0009 (5)
O3A	0.0656 (9)	0.0597 (8)	0.0334 (6)	0.0320 (7)	0.0109 (6)	−0.0008 (6)
O4A	0.0568 (8)	0.0485 (7)	0.0289 (6)	0.0187 (6)	0.0043 (6)	−0.0008 (5)
C1A	0.0396 (9)	0.0387 (8)	0.0277 (7)	0.0068 (7)	0.0083 (6)	0.0003 (6)
C2A	0.0420 (9)	0.0428 (8)	0.0262 (7)	0.0128 (8)	0.0073 (6)	0.0014 (6)
C3A	0.0401 (8)	0.0367 (7)	0.0288 (7)	0.0081 (6)	0.0068 (7)	−0.0011 (6)
C4A	0.0397 (8)	0.0421 (8)	0.0274 (7)	0.0105 (8)	0.0056 (6)	−0.0012 (6)
C5A	0.0383 (8)	0.0351 (7)	0.0298 (7)	0.0065 (7)	0.0056 (6)	−0.0012 (6)
N1A	0.0506 (9)	0.0441 (8)	0.0422 (8)	0.0090 (7)	0.0148 (7)	−0.0026 (6)
N2A	0.0533 (10)	0.0551 (9)	0.0386 (8)	0.0173 (8)	0.0036 (7)	−0.0042 (7)
C6A	0.0620 (12)	0.0484 (10)	0.0347 (8)	0.0055 (10)	0.0071 (8)	−0.0039 (7)
C7A	0.0564 (12)	0.0457 (9)	0.0334 (8)	0.0097 (9)	0.0029 (8)	−0.0012 (7)
C8A	0.0392 (8)	0.0369 (8)	0.0362 (8)	0.0018 (7)	0.0048 (7)	−0.0005 (6)
C9A	0.0458 (10)	0.0465 (9)	0.0261 (7)	0.0040 (7)	0.0044 (7)	0.0019 (6)
C10A	0.0456 (10)	0.0477 (9)	0.0375 (8)	0.0034 (8)	0.0102 (7)	0.0027 (7)
O1B	0.0608 (8)	0.0608 (8)	0.0340 (6)	0.0342 (7)	0.0042 (6)	0.0021 (6)
O2B	0.0653 (9)	0.0533 (7)	0.0293 (6)	0.0242 (7)	0.0094 (6)	0.0018 (5)
O3B	0.0653 (9)	0.0577 (8)	0.0353 (6)	0.0334 (7)	0.0073 (6)	0.0009 (6)
O4B	0.0659 (9)	0.0579 (8)	0.0288 (6)	0.0227 (7)	−0.0008 (6)	−0.0005 (5)
C1B	0.0407 (9)	0.0373 (8)	0.0301 (7)	0.0084 (7)	0.0087 (7)	0.0018 (6)
C2B	0.0406 (9)	0.0380 (8)	0.0281 (7)	0.0122 (7)	0.0063 (6)	0.0011 (6)
C3B	0.0401 (8)	0.0344 (7)	0.0273 (7)	0.0066 (6)	0.0054 (7)	−0.0005 (6)
C4B	0.0398 (8)	0.0389 (7)	0.0279 (7)	0.0095 (7)	0.0032 (6)	−0.0035 (6)
C5B	0.0387 (9)	0.0343 (7)	0.0321 (7)	0.0076 (7)	0.0019 (6)	−0.0010 (6)
N1B	0.0458 (9)	0.0450 (8)	0.0424 (8)	0.0103 (7)	0.0030 (7)	0.0035 (6)
N2B	0.0553 (10)	0.0565 (9)	0.0368 (8)	0.0171 (8)	0.0105 (7)	0.0065 (7)
C6B	0.0468 (10)	0.0480 (9)	0.0368 (9)	0.0012 (9)	0.0078 (7)	−0.0046 (7)
C7B	0.0461 (10)	0.0455 (9)	0.0278 (7)	0.0047 (8)	0.0048 (7)	−0.0013 (6)
C8B	0.0406 (9)	0.0390 (8)	0.0342 (8)	0.0033 (7)	0.0095 (7)	0.0019 (6)
C9B	0.0577 (12)	0.0480 (9)	0.0330 (8)	0.0116 (9)	0.0093 (8)	−0.0003 (7)
C10B	0.0588 (12)	0.0554 (10)	0.0325 (9)	0.0105 (10)	0.0062 (8)	0.0045 (8)

### Geometric parameters (Å, °)

**Table:**

O1A—C1A	1.299 (2)	O1B—C1B	1.2823 (19)
O1A—H1O1	0.8903	O2B—C1B	1.2303 (19)
O2A—C1A	1.2194 (18)	O3B—C5B	1.310 (2)
O3A—C5A	1.269 (2)	O3B—H1O3	0.9176
O4A—C5A	1.2423 (18)	O4B—C5B	1.2133 (19)
C1A—C2A	1.511 (2)	C1B—C2B	1.511 (2)
C2A—C3A	1.518 (2)	C2B—C3B	1.516 (2)
C2A—H2AA	0.9700	C2B—H2BA	0.9700
C2A—H2AB	0.9700	C2B—H2BB	0.9700
C3A—C4A	1.516 (2)	C3B—C4B	1.521 (2)
C3A—H3AA	0.9700	C3B—H3BA	0.9700
C3A—H3AB	0.9700	C3B—H3BB	0.9700
C4A—C5A	1.511 (2)	C4B—C5B	1.505 (2)
C4A—H4AA	0.9700	C4B—H4BA	0.9700
C4A—H4AB	0.9700	C4B—H4BB	0.9700
N1A—C10A	1.333 (2)	N1B—C10B	1.338 (2)
N1A—C6A	1.342 (2)	N1B—C6B	1.345 (2)
N1A—H1AA	0.8600	N1B—H1BA	0.8600
N2A—C8A	1.323 (2)	N2B—C8B	1.320 (2)
N2A—H2AC	0.8600	N2B—H2BC	0.8600
N2A—H2AD	0.8600	N2B—H2BD	0.8600
C6A—C7A	1.357 (3)	C6B—C7B	1.368 (3)
C6A—H6AA	0.9300	C6B—H6BA	0.9300
C7A—C8A	1.422 (2)	C7B—C8B	1.403 (2)
C7A—H7AA	0.9300	C7B—H7BA	0.9300
C8A—C9A	1.407 (2)	C8B—C9B	1.415 (2)
C9A—C10A	1.370 (3)	C9B—C10B	1.357 (3)
C9A—H9AA	0.9300	C9B—H9BA	0.9300
C10A—H10A	0.9300	C10B—H10B	0.9300
			
C1A—O1A—H1O1	120.0	C5B—O3B—H1O3	117.8
O2A—C1A—O1A	120.78 (15)	O2B—C1B—O1B	121.54 (15)
O2A—C1A—C2A	122.33 (15)	O2B—C1B—C2B	121.05 (14)
O1A—C1A—C2A	116.89 (13)	O1B—C1B—C2B	117.40 (13)
C1A—C2A—C3A	115.41 (12)	C1B—C2B—C3B	114.68 (13)
C1A—C2A—H2AA	108.4	C1B—C2B—H2BA	108.6
C3A—C2A—H2AA	108.4	C3B—C2B—H2BA	108.6
C1A—C2A—H2AB	108.4	C1B—C2B—H2BB	108.6
C3A—C2A—H2AB	108.4	C3B—C2B—H2BB	108.6
H2AA—C2A—H2AB	107.5	H2BA—C2B—H2BB	107.6
C4A—C3A—C2A	110.61 (12)	C2B—C3B—C4B	110.07 (12)
C4A—C3A—H3AA	109.5	C2B—C3B—H3BA	109.6
C2A—C3A—H3AA	109.5	C4B—C3B—H3BA	109.6
C4A—C3A—H3AB	109.5	C2B—C3B—H3BB	109.6
C2A—C3A—H3AB	109.5	C4B—C3B—H3BB	109.6
H3AA—C3A—H3AB	108.1	H3BA—C3B—H3BB	108.2
C5A—C4A—C3A	115.55 (13)	C5B—C4B—C3B	115.13 (13)
C5A—C4A—H4AA	108.4	C5B—C4B—H4BA	108.5
C3A—C4A—H4AA	108.4	C3B—C4B—H4BA	108.5
C5A—C4A—H4AB	108.4	C5B—C4B—H4BB	108.5
C3A—C4A—H4AB	108.4	C3B—C4B—H4BB	108.5
H4AA—C4A—H4AB	107.5	H4BA—C4B—H4BB	107.5
O4A—C5A—O3A	122.35 (15)	O4B—C5B—O3B	120.63 (15)
O4A—C5A—C4A	119.61 (14)	O4B—C5B—C4B	122.68 (15)
O3A—C5A—C4A	118.04 (13)	O3B—C5B—C4B	116.69 (13)
C10A—N1A—C6A	120.06 (16)	C10B—N1B—C6B	120.21 (16)
C10A—N1A—H1AA	120.0	C10B—N1B—H1BA	119.9
C6A—N1A—H1AA	120.0	C6B—N1B—H1BA	119.9
C8A—N2A—H2AC	120.0	C8B—N2B—H2BC	120.0
C8A—N2A—H2AD	120.0	C8B—N2B—H2BD	120.0
H2AC—N2A—H2AD	120.0	H2BC—N2B—H2BD	120.0
N1A—C6A—C7A	122.55 (17)	N1B—C6B—C7B	120.57 (17)
N1A—C6A—H6AA	118.7	N1B—C6B—H6BA	119.7
C7A—C6A—H6AA	118.7	C7B—C6B—H6BA	119.7
C6A—C7A—C8A	118.83 (17)	C6B—C7B—C8B	120.38 (16)
C6A—C7A—H7AA	120.6	C6B—C7B—H7BA	119.8
C8A—C7A—H7AA	120.6	C8B—C7B—H7BA	119.8
N2A—C8A—C9A	120.67 (16)	N2B—C8B—C7B	120.90 (15)
N2A—C8A—C7A	122.04 (16)	N2B—C8B—C9B	121.69 (16)
C9A—C8A—C7A	117.29 (16)	C7B—C8B—C9B	117.41 (16)
C10A—C9A—C8A	119.73 (16)	C10B—C9B—C8B	118.75 (17)
C10A—C9A—H9AA	120.1	C10B—C9B—H9BA	120.6
C8A—C9A—H9AA	120.1	C8B—C9B—H9BA	120.6
N1A—C10A—C9A	121.52 (17)	N1B—C10B—C9B	122.66 (17)
N1A—C10A—H10A	119.2	N1B—C10B—H10B	118.7
C9A—C10A—H10A	119.2	C9B—C10B—H10B	118.7
			
O2A—C1A—C2A—C3A	8.1 (3)	O2B—C1B—C2B—C3B	−4.8 (3)
O1A—C1A—C2A—C3A	−172.34 (16)	O1B—C1B—C2B—C3B	175.08 (17)
C1A—C2A—C3A—C4A	175.44 (15)	C1B—C2B—C3B—C4B	−175.99 (14)
C2A—C3A—C4A—C5A	174.42 (15)	C2B—C3B—C4B—C5B	−176.94 (15)
C3A—C4A—C5A—O4A	6.7 (3)	C3B—C4B—C5B—O4B	−5.5 (3)
C3A—C4A—C5A—O3A	−173.38 (17)	C3B—C4B—C5B—O3B	175.12 (16)
C10A—N1A—C6A—C7A	−0.6 (3)	C10B—N1B—C6B—C7B	−0.9 (3)
N1A—C6A—C7A—C8A	−0.3 (3)	N1B—C6B—C7B—C8B	0.0 (3)
C6A—C7A—C8A—N2A	−178.87 (18)	C6B—C7B—C8B—N2B	−178.91 (18)
C6A—C7A—C8A—C9A	1.1 (3)	C6B—C7B—C8B—C9B	1.0 (3)
N2A—C8A—C9A—C10A	178.99 (18)	N2B—C8B—C9B—C10B	178.79 (18)
C7A—C8A—C9A—C10A	−1.0 (3)	C7B—C8B—C9B—C10B	−1.1 (3)
C6A—N1A—C10A—C9A	0.8 (3)	C6B—N1B—C10B—C9B	0.8 (3)
C8A—C9A—C10A—N1A	0.1 (3)	C8B—C9B—C10B—N1B	0.3 (3)

### Hydrogen-bond geometry (Å, °)

**Table:**

*D*—H···*A*	*D*—H	H···*A*	*D*···*A*	*D*—H···*A*
O1A—H1O1···O1B^i^	0.89	1.57	2.4591 (17)	172
N1A—H1AA···O1B^ii^	0.86	2.59	3.183 (2)	127
N1A—H1AA···O2B^ii^	0.86	1.87	2.729 (2)	177
N2A—H2AC···O2A^iii^	0.86	2.18	2.9627 (19)	151
N2A—H2AD···O4B	0.86	2.10	2.948 (2)	170
O3B—H1O3···O3A^iv^	0.92	1.56	2.4791 (17)	176
C10A—H10A···O4B^v^	0.93	2.55	3.161 (2)	124
C7A—H7AA···O3B	0.93	2.47	3.370 (2)	162

Symmetry codes: (i) *x*−1, *y*+1, *z*−1; (ii) *x*−1, *y*+1, *z*; (iii) *x*, −*y*+3/2, *z*+1/2; (iv) *x*, *y*, *z*+1; (v) −*x*+1, *y*+1/2, −*z*+3/2.
